# UMBILICAL AND EPIGASTRIC HERNIA REPAIR: A SYSTEMATIC REVIEW

**DOI:** 10.1590/0102-6720202400014e1807

**Published:** 2024-06-17

**Authors:** José Roberto ALVES, Luis Felipe Mondardo SPENGLER, Leonardo Busch JUSTINO, Gustavo Busch JUSTINO, Iago Koerich SILVA, Enio Campos AMICO

**Affiliations:** 1 Universidade Federal de Santa Catarina, Department of Surgery – Florianópolis (SC), Brazil; 2 Universidade Federal do Rio Grande do Norte, Department of Surgery – Natal (RN), Brazil.

**Keywords:** Umbilical Hernia, Ventral Hernia, Abdominal Wall, General Surgery, Hérnia Umbilical, Hérnia Ventral, Parede Abdominal, Cirurgia Geral

## Abstract

**BACKGROUND:**

Umbilical and epigastric hernias are among the most common hernias of the abdominal wall; however, there is a lack of standardization for their treatment.

**AIMS:**

To clarify the controversies regarding therapeutic possibilities, indications, and surgical techniques for umbilical and epigastric hernia repair.

**METHODS:**

A systematic review and qualitative analysis of randomized clinical trials published in the last 20 years, involving adults (aged 18 years and over) with umbilical and/or epigastric hernias, was performed by systematically searching the PubMed/Medline, Cochrane, SciELO, and LILACS databases. The risk of bias in individual studies was assessed using the Cochrane Risk of Bias Tool.

**RESULTS:**

Initially, 492 studies were selected and, subsequently, 15 randomized controlled clinical trials were chosen that met the inclusion criteria and underwent full reading and qualitative analysis, considering possible bias.

**CONCLUSIONS:**

This review concluded that it is evident the superiority of the use of meshes in the repair of epigastric/primary umbilical hernias with a defect larger than 1 cm, even in certain emergency situations. However, suture repair is a good option for patients with a defect smaller than 1 cm. In the laparoscopic approach, recent evidence points towards possible superiority in fixation with fibrin sealant, and fascial defect closure is recommended. In addition, due to a scarcity of randomized controlled trials with low risk of bias, further studies are needed on types, positioning and fixation techniques, as well as the real role of video-assisted laparoscopic surgery in the correction of hernias, especially umbilical.

## RESUMO

**RACIONAL:**

As hérnias umbilicais e epigástricas estão entre as hérnias mais comuns da parede abdominal, porém falta padronização em relação ao seu tratamento.

**OBJETIVOS:**

Esclarecer as controvérsias acerca das possibilidades terapêuticas, indicações e técnicas cirúrgicas relacionadas ao tratamento das hérnias umbilicais e epigástricas.

**MÉTODOS:**

Foi realizada revisão sistemática e análise qualitativa dos estudos publicados nos últimos 20 anos, envolvendo indivíduos maiores de 18 anos com hérnias umbilicais e/ou epigástricas, por meio de busca nas bases de dados PubMed/Medline, Cochrane, LILACS e SciELO, sendo selecionados estudos do tipo ensaios clínicos randomizados controlados para análise. Foi determinado o risco de *viés* de cada estudo individualmente de acordo com a ferramenta Cochrane *Risk of Bias Tool*.

**RESULTADOS:**

Foram inicialmente selecionados 492 estudos e, posteriormente, escolhidos 15 ensaios clínicos randomizados controlados que preencheram os critérios de inclusão e foram submetidos à leitura integral e análise qualitativa, considerando os possíveis *viés*.

**CONCLUSÕES:**

Esta revisão concluiu que é evidente a superioridade do uso de telas no tratamento das hérnias epigástricas/umbilicais primárias com defeito superior a 1 cm, mesmo em certas situações de emergência. No entanto, o reparo com sutura pode ser uma opção em pacientes com defeito menor que 1 cm. Na abordagem laparoscópica, evidências recentes apontam para possível superioridade na fixação com selantes de fibrina e o fechamento do defeito fascial é recomendado. Ademais, devido à escassez de ensaios clínicos randomizados controlados com baixo risco de *viés*, foi identificada a necessidade de desenvolver mais estudos sobre os tipos, técnicas de posicionamento e fixação das telas, assim como sobre qual o real papel da cirurgia videolaparoscópica na correção das hérnias, especialmente as umbilicais.

## INTRODUCTION

Abdominal wall hernias have a multifactorial etiology, such as smoking, collagen disorders, aging process, and congenital abdominal wall defects, among others, and represent a set of high prevalence diseases. They are often presented clinically with bulging and/or local pain, particularly during physical exertion, which impacts patients’ quality of life to different extents^17^. Umbilical and epigastric hernias, in turn, constitute an important portion of this set with a prevalence of, approximately, 2% in adults worldwide, being responsible for more than 200,000 surgeries per year in the United States of America^13,20^.

The first report of surgical treatment of umbilical or epigastric hernias was dated 1740, and since then, with the introduction of the Mayo repair technique approximately a century ago, therapeutic modalities have evolved focusing mainly on the reduction of recurrence rates and time away from work^13,17^.

Historically, recurrence rates varied between 30 and 40% before the introduction of mesh repair and decreased significantly after it, about 5%^24^. However, although the new techniques reduced the time away from work, due to less invasive interventions and lower complication rates, they progressively raised surgical costs in the last decades^13^.

Moreover, there are numerous controversies and absence of standardization concerning the treatment of umbilical and epigastric hernias. The state-of-the-art approach is still unknown, for example, in terms of the best suture techniques (continuous or interrupted), number of layers, mesh indications, surgery contraindications, and when to decide on a different method from the conventional. Since these decisions depend on specific aspects, such as individual characteristics, comorbidities, hernia size, surgeon experience, availability of resources, and the expectations of those involved in the procedure, literature research is fundamental^14^.

Therefore, this systematic review aims to clarify the main controversies and recommendations regarding the current treatment of umbilical and/or epigastric hernias based on the best available evidence.

## METHODS

This systematic review was registered in the International Prospective Register of Systematic Reviews (PROSPERO) platform (https://www.crd.york.ac.uk/prospero/display_record.php?ID=CRD42020192450) and conducted according to recommendations from Preferred Reporting Items for Systematic Reviews and Meta-Analyses (PRISMA) protocol^12^. We systematically searched for studies published in Portuguese, Spanish, and English in the last 20 years in the Medical Literature Analysis and Retrieval System Online (PubMed/Medline), Cochrane Central Register of Controlled Trials (CENTRAL), Scientific Electronic Library On-line SciELO, and Latin American and Caribbean Health Sciences Literature (LILACS). The search strategy used was the *Boolean* terms and operators: “hernia” [Title] AND (“umbilical” [Title] OR “epigastric” [Title]) OR Hernia, Umbilical [MeSH Terms]. Randomized controlled trials (RCTs) conducted with adult individuals (aged 18 years or older) and with a sample of 40 or more participants were selected. All studies were independently and thoroughly reviewed by two authors using Rayyan organizing platform support to facilitate the exclusion of repeated studies and those that did not match the pre-defined inclusion criteria^16^. Disagreements were resolved by consensus among the two authors. Studies that included incisional hernias or other types of ventral hernias in their samples, besides cirrhotic participants or pregnant women, were excluded.

Data from all studies were analyzed to elucidate current controversies concerning the repair of umbilical and/or epigastric hernias. Finally, we extracted information about sample size, type of study, location and size of hernias, repair technique, surgery duration and time until discharge, recurrence rate, early and late postoperative complications (qualification and identification of occurrence/incidence rate), and mortality rate.

The European Hernia Society and the Americas Hernia Society Guidelines for treatment of umbilical and epigastric hernias and Costa et al.^7^ study (even though they were studies with criteria outside those established in the search strategy of this systematic review) were included to compare and complement some topics not approached by the selected RCTs, as well as improve and expand the discussion of topics related to the treatment of umbilical and epigastric hernias^7,9^.

Quality assessment and risk of bias of individual RCTs were analyzed with the Cochrane Risk of Bias Tool. Each study received a score of high, moderate, or low risk of bias.

## RESULTS

Applying the above-mentioned search strategy, 492 studies were initially found. After the exclusion of duplicates and the application of inclusion and exclusion criteria, 15 RCTs were selected. The European Hernia Society and the Americas Hernia Society Guidelines and Costa et al.^7^ study were also included in the discussion as mentioned above^7,9^.

**Figure 1:**
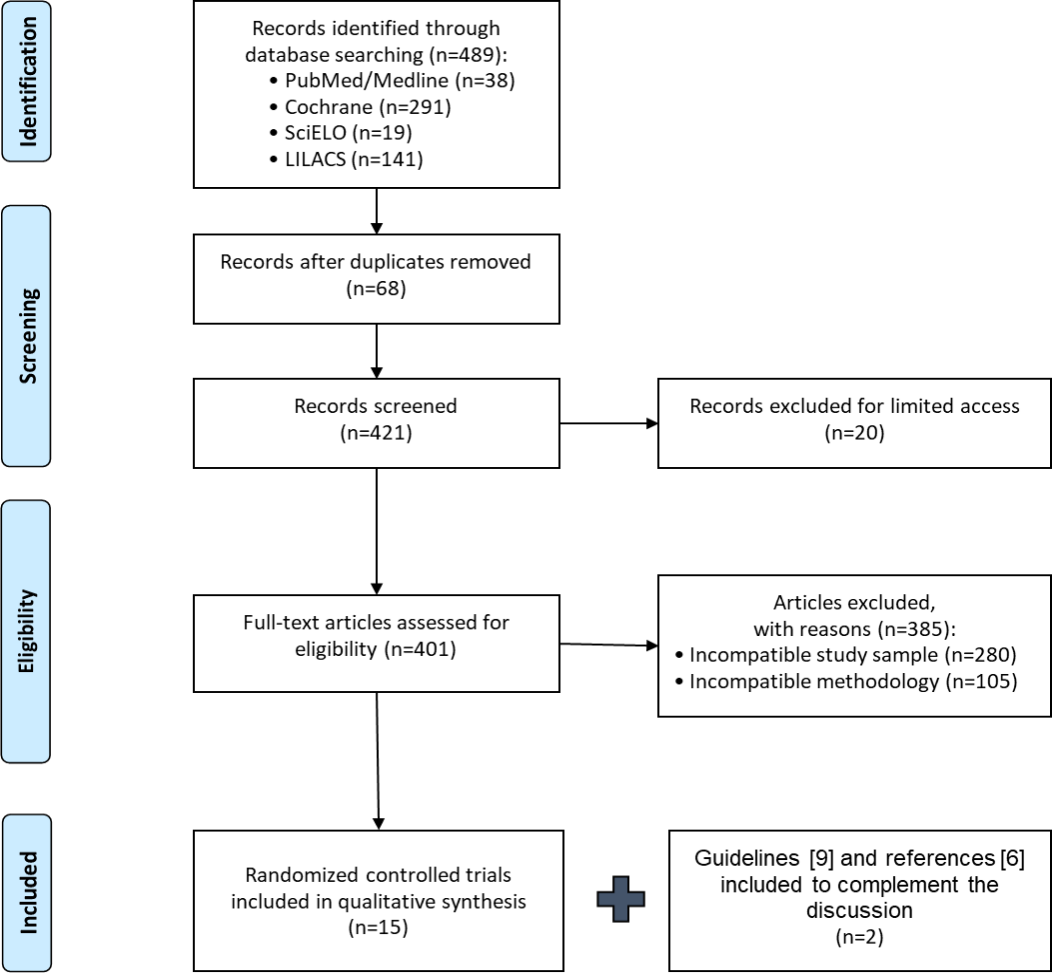
Preferred Reporting Items for Systematic Reviews and Meta-Analyses (PRISMA) flowchart of study selection.

Most of the excluded studies either did not address the subjects of interest or were not eligible RCTs according to the inclusion/exclusion criteria (Figure 1). The risk of bias of the 15 selected RCTs was classified according to the Cochrane Risk of Bias Tool (Table 1).

**Table 1:** Fifteen eligible studies (randomized controlled trials) bias assessment according to Cochrane Risk of Bias Tool.

Studies	Random sequence generation	Allocation concealment	Selective reporting	Blinding participants	Blinding outcome assessment	Incomplete outcome data	Other sources of bias
Kaufmann et al.^10^	Low risk	Low risk	Low risk	Low risk	Low risk	Low risk	Low risk
Arroyo et al.^3^	Low risk	Low risk	Low risk	High risk	High risk	Low risk	Low risk
Ponten et al.^19^	Low risk	Low risk	Low risk	Low risk	Low risk	Low risk	Low risk
Abdel-Baki et al.^1^	Low risk	High risk	Low risk	High risk	High risk	Low risk	Low risk
Christoffersen et al.^5^	Low risk	Low risk	Low risk	High risk	Low risk	Low risk	Low risk
Malik^11^	Low risk	Low risk	Low risk	High risk	High risk	Low risk	Low risk
Polat et al.^18^	Low risk	High risk	Low risk	High risk	High risk	High risk	High risk
Bessa et al.^4^	Low risk	Low risk	Low risk	High risk	High risk	Low risk	Low risk
Eriksen et al.^8^	Low risk	Low risk	Low risk	Low risk	Low risk	Low risk	Low risk
Pietro Díaz et al.^21^	Low risk	Low risk	Low risk	High risk	Low risk	Low risk	Low risk
Purushotham and Madhu^22^	Low risk	High risk	Low risk	High risk	High risk	Low risk	Low risk
Abo-Ryia et al.^2^	Low risk	High risk	Low risk	High risk	High risk	Low risk	Low risk
Tunio^23^	Low risk	Low risk	Low risk	High risk	High risk	Low risk	Low risk
Othman et al.^15^	Low risk	High risk	Low risk	High risk	High risk	Low risk	Low risk
Christoffersen et al.^6^	Low risk	Low risk	Low risk	Low risk	Low risk	Low risk	Low risk

This table shows four studies classified as low risk of bias in all domains^2,6,10,17^

One study was classified as high risk of bias in five domains and four other studies as high risk of bias in three domains^1,18,19,20,22^. These RCTs presented their major problems related to the blinding bias of participants and evaluators. Thus, the conclusions of these studies were carefully analyzed and weighted in the discussion, as well as the inferences that originated from the studies with low risk of bias.

The main characteristics and outcomes of the selected RCTs are presented in Table 2.

**Table 2:** Disclosure of 15 eligible randomized controlled trials main outcomes related to the treatment of umbilical and epigastric hernias.

Studies	Type of hernia included	Selected theme	N	Intervention group	Control group	Criteria for mesh indication	Statistically significant outcome
Kaufmann et al.^10^	Umbilical	Mesh use	300	Mesh repair	Suture repair	Defect between 1 and 4 cm	Fewer recurrence rate within two years in the mesh group (3.6% vs 11.4%)
Arroyo et al.^3^	Umbilical	Mesh use	200	Mesh repair	Suture repair	Defect above 3 cm	Fewer recurrence rate within five years in the mesh group (1% vs 11%)
Ponten et al.^19^	Umbilical and epigastric	Mesh use	348	Mesh repair	PVP repair	Defect wider than 2 fingers’ width	Fewer reoperation rate (4.0% vs 10.7%) and local complications rates (22.1% vs 32.5%) in the mesh group
Abdel-Baki et al.^1^	Umbilical (acute incarcerated)	Mesh use	42	Mesh repair	Suture repair	Any size defect	Fewer recurrence rate within one and a half year in the mesh group (0% vs 19%)
Tunio^23^	Umbilical	Mesh use	86	Mesh repair	Suture repair	Defect above 3 cm	Fewer local complications, post-operative pain, recurrence rate within three years (2.3% vs 7.0%) and hospitalization days (4.30 days vs 6.14) in the mesh group
Bessa et al.^4^	Umbilical	Mesh position	80	Pre-peritoneal position	Pre-aponeurotic position	Defect between 4 and 10 cm	There were no significantly distinct outcomes between different positions
Abo-Ryia et al.^2^	Umbilical	Mesh position	60	Retrorectal position	Pre-aponeurotic position	Any size defect	Retrorectal position was associated with fewer post-operative pain rate (VAS score of 4.87 vs 7.07)
Eriksen et al.^8^	Umbilical	Mesh fixation	40	Fixation with fibrin sealant	Fixation with titanium grapples	Defect between 1.5 and 5.0 cm	Fixation with fibrin sealant was associated with fewer post-operative pain rate (VAS score of 1.9 vs 4.7)
Malik^11^	Umbilical	Laparoscopic surgery	337	Laparoscopic repair	Open repair	Any size defect	Fewer hematoma rate (1.61% vs 23.64%), seroma rate (4.03% vs 11.48%), chronic pain rate (2.41% vs 8.7%), and hospitalization days (2 vs 5) in the laparoscopic group
Purushotham et al.^22^	Umbilical	Laparoscopic surgery	42	Laparoscopic repair	Open repair	Any size defect	Fewer post-op pain rate (VAS score of 3.05 vs 7.48) and hospitalization days (2 vs 4) in the laparoscopic group
Othman et al.^15^	Umbilical	Laparoscopic surgery	40	Laparoscopic repair	Open repair	Any size defect	Fewer post-operative pain rate in the laparoscopic group (VAS score of 2.76 vs 4.73)
Christoffersen et al.^6^	Umbilical	Laparoscopic surgery	80	Fascial defect closure	Without fascial defect closure	Defect between 2 and 6 cm	Fewer than 2-year recurrence (13.88% vs 32.43%) and seroma (35% vs 55%) rate in closure group
Christoffersen et al.^5^	Umbilical and epigastric	Complication prevention	56	Abdominal binders use	Without abdominal binders	Defect between 2 and 8 cm	There were no significantly distinct outcomes in abdominal binder use
Polat et al.^18^	Umbilical	New techniques	50	PHS repair	Others techniques	Defect below 4 cm	Fewer post-op pain rate in the PHS group (McGill pain score of 0.6 vs 0.9)
Pietro Díaz et al.^21^	Umbilical	New techniques	82	Transumbilical incision	Infraumbilical incision	Any size defect	There were no significantly distinct outcomes between different incision types

N: sample size; cm: centimeters; PVC: proceed ventral patch; PHS: Prolene Hernia System; VAS: Visual Analog Scale.

## DISCUSSION

Despite the low quality of evidence (absence of RCT studies), international guidelines recommend that diagnosis should be based on clinical examination, and imaging should be considered only in dubious presentations^9^. In these cases, ultrasound or computed tomography (without intravenous contrast) are the best diagnostic tools, and a useful option is the Valsalva Maneuver during the exam^9^.

The use of mesh in the treatment of inguinal and incisional hernias is already well established. However, this technique is not popular yet among primary ventral hernias, especially in those with smaller dimensions, despite strong evidence weighting towards it^9^.

Two RCTs showed lower recurrence rates and the need for reoperations of umbilical and epigastric hernias when mesh use was opted for, especially in defects greater than 3–4 cm^1,21^. One RCT with a low risk of bias identified these same benefits in hernias equal to or larger than 1 cm^9^. Another study demonstrated a shorter hospital stay and earlier return to work activities in patients with defects greater than 3 cm who underwent mesh repair^21^. None of the RCTs demonstrated a significantly higher rate of local complications related to the use of mesh^1,9,21^.

In the context of emergency repair due to acute complications, one RCT demonstrated low recurrence rate for mesh repair^1^. The presence of ischemia or intestinal necrosis, which demands intestinal resection, was not considered a contraindication for mesh repair (including microporous mesh) in cases of strangulated umbilical hernias, except when there was gross contamination of the surgical site or signs of generalized peritonitis^1^. However, this information should be viewed with caution since it was extracted from a high risk of bias RCT (related to concealment of the allocation, blinding of participants and assessors)^1^.

There is no evidence-based information in the literature comparing different techniques regarding mesh placement, such as the requirement of a minimal mesh overlap in relation to hernia margins^1,5,10^. However, studies with different methodologies established a 3 cm minimum overlap in all margins as a recommended standard^1,5,10^.

In addition, the fixation and type of mesh used in open repair were not compared in eligible RCTs. Nevertheless, most of them opted for an inorganic polypropylene monofilament mesh fixed by interrupted unabsorbable sutures, mostly Prolene 0 (zero), as a standard technique^1,4,5,10,20^. A retrospective observational study suggested that newer prosthetics design, such as malleable and light mesh, allows an easier and faster mesh positioning and fixation, favoring the surgical procedure^10^.

Regarding the use of three-dimensional mesh, one RCT compared the Prolene Hernia System (PHS) to other techniques demonstrating low postoperative pain rates, but it was associated with a high risk of bias^18^. Therefore, although being an apparently promising technique, it lacks a more robust proof of its real advantages in the therapeutic scenario of umbilical and epigastric hernias^18^.

Regarding the position adopted in the placement of the mesh in relation to the aponeurosis, there is no strong evidence in the literature with a low risk of bias that demonstrates the superiority of any position^4^. A small RCT presented possible superiority of the retrorectal position or Rives-Stoppa technique^2^. Nevertheless, its results should be viewed with caution, since it is an unblinded study that included only 60 participants, presenting a high risk of bias^2^. In addition, most RCTs used the pre-aponeurotic positioning technique as standard^1,4,5,9,19^. Therefore, this is a subject that needs further investigation to obtain a more formal recommendation, based on greater evidence. For the time being, mainly due to greater technical simplicity, the pre-aponeurotic position remains an adequate option for mesh placement.

The need to enlarge the hernia defect to ensure a satisfactory overlap of the mesh is also a matter of controversy. Evidence from an RCT states that the enlargement of a defect is not related to worse results^5^. Furthermore, high-quality evidence with a low global risk of bias recommends that all patients with 1 cm umbilical hernias or greater, should undergo repair with the use of mesh regardless of their characteristics (body mass index [BMI], age, smoke status, sedentariness, etc.)^1,10,21^.

In umbilical and epigastric hernia defects smaller than 1 cm, the decision of placement of mesh should be individualized, since no RCT included this characteristic. A retrospective observational study, however, states that satisfactory results may be achieved regardless of the use of mesh, with similar safety and efficacy endpoints between both approaches^9^. Techniques without the use of mesh are recommended especially in non-smoking and immunocompetent patients with defects smaller than 1 cm and a good vitality aponeurosis, a statement based only in observational studies^9^.

The best approach to hernia repair without the use of mesh, such as primary tissue repair through continuous sutures, interrupted stitches, and Mayo repair, is still unclear since no RCT of umbilical/epigastric hernias evaluating these aspects was found. However, observational studies showed a 10% lower recurrence rate when Mayo repair was opted among the techniques without the use of mesh, and thus this procedure may be an adequate choice in hernia defects smaller than 1 cm, as described previously^9^.

Video-assisted laparoscopic surgery of abdominal wall hernias became popular in recent years, being suggested by some studies as a possible standard treatment for primary large ventral hernias^9^. However, only retrospective observational studies and RCTs with a considerable risk of bias proposed the laparoscopic approach in special circumstances, for example, umbilical and epigastric hernias associated with multiple simultaneous defects in the abdominal wall and defects larger than 4 cm, especially in obese patients^9,11,19^. In these situations, the laparoscopic approach indicated a lower risk of postoperative pain and length of hospitalization^9,11,15^.

It is also known that the use of mesh with at least one side made of non-adhesive material is required to perform the video-assisted laparoscopic repair to avoid intestinal loops adhesions with the mesh surface, increasing surgery-related cost^10,15^. The comparison between the types of mesh adopted in video-assisted laparoscopic repairs was not the scope of any RCT. However, polytetrafluoroethylene mesh was the most frequently used in the studies included in this review^11,19^. Regarding the technique of fixing these mesh through laparoscopy, strong evidence suggests that the use of fibrin-based sealants was superior concerning the evaluation of postoperative pain intensity in comparison with titanium clips^8^. The closure of the fascial defect in these cases was also associated with less seroma formation and long-term recurrence^23^.

Moreover, this review has not yet identified any RCT with a low risk of bias in all categories that could demonstrate the superiority of the laparoscopic approach over the conventional (open) approach in hernias of any size defect, and, hence, this may be a possible subject to inspire future studies^11,15^. Therefore, based on the best available evidence, it is still early to establish laparoscopy as a “gold standard” access route for umbilical hernia repair^11,22^.

The surgeon’s experience should be considered when deciding the best type of access to the surgical site since there is a learning curve related to laparoscopy repair (more than 30 procedures)^9,11,22^. Furthermore, epigastric hernias were not included in any of the studies that evaluated this subject, and therefore, the surgeon’s experience becomes a more important factor regarding the decision of the most appropriate access route in these cases.

The indication for conservative treatment was not assessed in any RCT. However, other methodologically different studies evaluated this hypothesis and stated, based on clinical observation, that conservative treatment with follow-up is safe in asymptomatic patients with hernia defects smaller than 1 cm^9^. This was evidenced especially in individuals who did not notice the hernia spontaneously since the chance of it becoming symptomatic in 5 years of follow-up and requiring surgical repair will be 16% in elective conditions and 4% in emergency conditions^9^. It is also necessary to note that patients with high surgical risk were not assessed by any of the studies identified in this review. Therefore, as the general complication rates of these hernias are between 4 and 12%, conservative treatment may be considered in these cases^9^. However, it is worth reaffirming that this is not an RCT-based recommendation.

The use of abdominal binders in the postoperative period was the main objective of only one RCT, but it was not possible to demonstrate any benefit of this recommendation in patients with hernia size defect between 2 and 8 cm, especially regarding reduction rates of postoperative complications (recurrence, seromas, hematomas, among others)^5^.

A single dose of antibiotic prophylaxis at the time of induction of anesthesia was considered the standard conduct for patients undergoing surgery (with or without the use of mesh) in some RCTs to prevent infectious complications^1,2,4,11,19^. The most commonly used antibiotics in elective situations were Cefazolin or Amoxicillin plus Clavulanate^2,4,11,19^. In addition, during emergencies, the standard was the association of a third-generation cephalosporin with Metronidazole^1^.

Finally, some RCTs used a single dose of low molecular weight heparin as preoperative prophylactic medication in obese patients (BMI over 30) undergoing surgical treatment of umbilical hernias, with or without the use of mesh^1,2,4^. Among these studies, only one RCT (with a high risk of bias in three evaluated variables; see Table 1) maintained the use of low molecular weight heparin in the first 48 postoperative hours in obese patients undergoing hernioplasty/herniorrhaphy due to emergency incarcerated/strangulated umbilical hernia^1^.

The present review is limited due to the scarce number of high-quality methodological studies researching umbilical and epigastric hernias, probably due to the heterogeneity and lack of a standardized surgical approach in this context, which impairs the actual recommendations. The decision towards not including a meta-analysis is precisely based on this limitation.

Furthermore, the long-term recurrence rate is probably the most important data regarding the choice of technique applied to the surgical correction of any type of hernia. However, studies with long-term follow-up are very scarce in the context of primary ventral hernias.

Robotic-assisted surgery research has grown in the past two decades in abdominal hernias and the advantages over traditional videolaparoscopy have been debated. Costa et al. analyzed the costs of a robotic program in a Brazilian public institution to treat abdominal hernias and concluded that the robotic system can increase intraoperative strategies, especially in complex hernias or incisional hernias, however, it adds a significant overall cost to traditional laparoscopic hernia repair, especially umbilical and epigastric hernias^7^.

This study differs from other methodologically similar articles and guidelines because it is constructed based only on RCTs, which is unprecedented regarding umbilical and epigastric hernias. It aimed to collect the best available recent evidence and exhibit the contrast between RCT-based recommendations and those based on observational studies to help the surgeon make the most judicious choice when treating primary ventral hernias.

The present study concluded that there is a scarce number of high-quality and low risk of bias RCTs evaluating surgical treatment of umbilical and/or epigastric hernias in the literature. This indication of mesh depends on the patient’s preferences and comorbidities and the surgeon’s experience, due to the lack of RCTs without a definitive answer when considering only these types of studies. On the other hand, although it is not a piece of information based on RCTs, the indication of herniorrhaphy without mesh or conservative treatment for cases of umbilical and epigastric hernias of less than 1 cm should be analyzed case-by-case^9^. Althoughcurrent evidence is weak, there seems to be a tendency to recommend repair by an open approach (laparotomy) for umbilical and epigastric hernias with defects of up to 4 cm^9,11,19^. Nevertheless, laparoscopic repair seems to be reserved for those cases with larger hernias, especially in obese patients with multiple simultaneous defects in the abdominal wall^9,11,19^. In these cases, recent evidence points towards a possible recommended closure of the fascial defect and mesh fixation with fibrin sealant^5,8^.

## CONCLUSIONS

High-quality evidence with a low global risk of bias recommends that all patients with umbilical hernias equal to or greater than 1 cm should undergo repair with the use of mesh regardless of their characteristics (BMI, age, smoke status, sedentariness, etc.), even during emergencies (incarcerated or strangulated hernias) in the absence of gross contamination or signs of generalized peritonitis. This technique provides a lower recurrence rate, overcoming any possible postoperative complications. The best composition, position, and fixation of the mesh as well as the ideal surgical access route are still unknown issues that need to be better investigated. Therefore, high-quality studies regarding the treatment of umbilical and epigastric hernias are warranted.
